# Development of monotonic neuronal tuning in the monkey inferotemporal cortex through long-term learning of fine shape discrimination

**DOI:** 10.1111/j.1460-9568.2010.07539.x

**Published:** 2011-02

**Authors:** Wataru Suzuki, Keiji Tanaka

**Affiliations:** 1 Cognitive Brain Mapping Laboratory, RIKEN Brain Science Institute2-1 Hirosawa, Wako, Saitama 351-0198, Japan; 2 Integrative Neural Systems Laboratory, RIKEN Brain Science InstituteHirosawa, Wako, Saitama, Japan; 3 Laboratory of Visual Physiology, National Institute of Sensory OrgansHigashigaoka, Meguro, Tokyo, Japan

**Keywords:** inferotemporal cortex, macaque, object recognition, plasticity, visual expertise

## Abstract

Visual expertise in discriminating fine differences among a group of similar objects can be obtained through extensive long-term training. Here we investigated the neural bases of this superior capability. The inferotemporal cortex, located at the final stage along the ventral visual pathway, was a candidate site in monkeys because cells there respond to various complex features of objects. To identify the changes that underlie the development of visual expertise in fine discrimination, we created a set of parametrically designed object stimuli and compared the stimulus selectivity of inferotemporal cells between two different training histories. One group of recordings was conducted after the monkeys had been extensively trained for fine discrimination (fine-discrimination period) and the other after the monkeys had been exposed only for coarse discrimination (coarse-discrimination period). We found that the tuning of responses recorded in the fine-discrimination period was more monotonic in the stimulus parameter space. The stimuli located at the extreme in the parameter space evoked the maximum responses in a larger proportion of cells and the direction of response decrease in the parameter space was more consistent. Moreover, the stimulus arrangement reconstructed from the responses recorded during the fine-discrimination period was more similar to the original stimulus arrangement. These results suggest that visual expertise could be based on the development, in the inferotemporal cortex, of neuronal selectivity monotonically tuned over the parameter space of the object images.

## Introduction

Visual expertise, or the extraordinary capability to discriminate fine differences among a group of similar object images, develops through extensive long-term training, and it is specific to the domain of stimuli used in the training. For example, experienced bird watchers expertly discriminate among bird species but not car models. Thus, the way of representing object images in the domain of one's expertise may be different from the general way of representing object images (Tanaka & Curran, 2001; Palmeri *et al.*, 2004; Tanaka *et al.*, 2005).

It has been shown that neurons in the anterior part of the monkey inferotemporal cortex selectively respond to complex object features (Logothetis & Sheinberg, 1996; Tanaka, 1996). It has also been suggested that response patterns over a population of inferotemporal cells represent object categories (Kiani *et al.*, 2007), whereas responses of individual inferotemporal cells fail to represent an object category (Vogels, 1999; Freedman *et al.*, 2003; De Baene *et al.*, 2008). Other studies have shown that long-term training of monkeys for discrimination among similar shapes results in an increase in the number of inferotemporal neurons that are responsive to these shapes (Logothetis *et al.*, 1995; Kobatake *et al.*, 1998), are selective among the shapes (Baker *et al.*, 2002) or are selective to the stimulus dimension relevant to the discrimination (Sigala & Logothetis, 2002; but also see De Baene *et al.*, 2008). Thus, neural substrates for the experience-dependent capability corresponding to visual expertise may exist in the inferotemporal cortex in monkeys (Gauthier *et al.*, 2000; but also see Grill-Spector *et al.*, 2004.

**Fig. 1:**
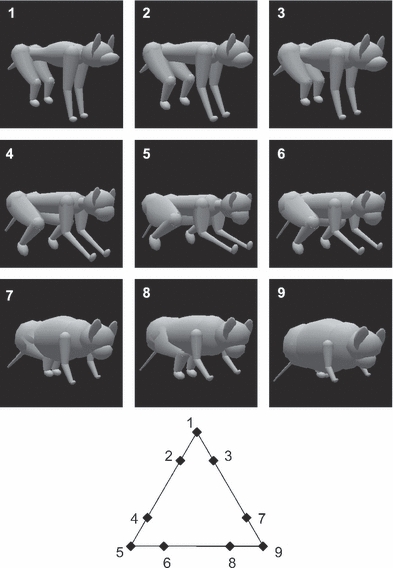
Object images used in the present study (top) and their configurations in the parameter space (bottom). The shapes of the objects were defined by 57 parameters, but the nine objects used were aligned on a two-dimensional hyper-plane. Three objects were located at the apices of a triangle on the hyper-plane and the other six were on the sides of the triangle, one-quarter of the distance away from an apex. Thus, objects fell into three groups composed of three similar objects.

The present study aimed to further reveal the neural bases of visual expertise. The results of previous monkey studies are suggestive but the studies were limited in that neuronal responses were recorded only after the training (Baker *et al.*, 2002; Sigala & Logothetis, 2002) or the stimuli were not parametrically designed (Kobatake *et al.*, 1998). In the present study, we used a set of parametrically designed shape stimuli (Fig. 1) and conducted single-cell recordings from the inferotemporal cortex in two different training histories: after fine-discrimination training and after experiencing only coarse discrimination. Various aspects of stimulus selectivity were compared between the two groups of inferotemporal neurons recorded in the two periods of recordings. We included the monotonicity of tuning in our analyses, because several recent studies have shown that inferotemporal cells tend to show monotonically tuned responses to a group of similar shapes (Kayaert *et al.*, 2005; Leopold *et al.*, 2006; De Baene *et al.*, 2007; Freiwald *et al.*, 2009). We found that the monotonic tuning was more prevalent after the fine-discrimination training.

## Materials and methods

### Subjects

Two male Japanese monkeys (*Macaca fuscata*), weighing 8.5 and 6.4 kg, participated in the experiments. The experimental plan was approved by the Experimental Animal Committee of RIKEN, and the monkeys were cared for in accordance with the National Institute of Health Guidelines for the Care and Use of Laboratory Animals and the ‘Guiding Principles of the Care and Use of Animals in the Field of Physiological Science’ of the Japanese Physiological Society.

### Stimuli, behavioral task and experimental design

The stimulus set used in this study has been described in detail previously (Suzuki *et al.*, 2006). Briefly, it consisted of nine animal-like objects characterized by 57 parameters. Each of the parameters defined a local shape parameter such as arm length, torso width, leg angle, etc. Although any of the 57 parameters changed from one object to another, the nine objects were located on a single two-dimensional hyper-plane in the parameter space spanned by the 57 parameters. Three objects were positioned at the apices of a triangle, and the other six were located on its sides at a quarter of the side length away from each apex (Fig. 1). This configuration resulted in a stimulus set consisting of three groups, each of which comprised three similar members. The images of objects seen from the same view point were used as stimuli. The largest dimension of the stimuli was around 6° in visual angle, and the brightest parts of the stimuli were 25 cd/m^2^. The background was 0.01 cd/m^2^.

Although we consistently used this set of object stimuli and the same task (see below) throughout the present experiments, the monkeys performed different levels of discrimination during different periods. In ‘coarse-discrimination’ trials, the monkeys discriminated stimuli only across groups, whereas they discriminated among members within a group in ‘fine-discrimination’ trials. In the ‘coarse-discrimination period’, the monkeys performed coarse discrimination only; they performed both fine and coarse discriminations in the other period (‘fine-discrimination period’). Blocks of coarse-discrimination trials were provided even during the fine-discrimination period, so as to maintain the motivation of the monkeys to perform the task and for the sake of the experimental design (see below). Trials of fine discrimination were not intermixed with those of coarse discrimination, but they were separated in different blocks. A block was composed of around 100 trials.

**Fig. 2:**
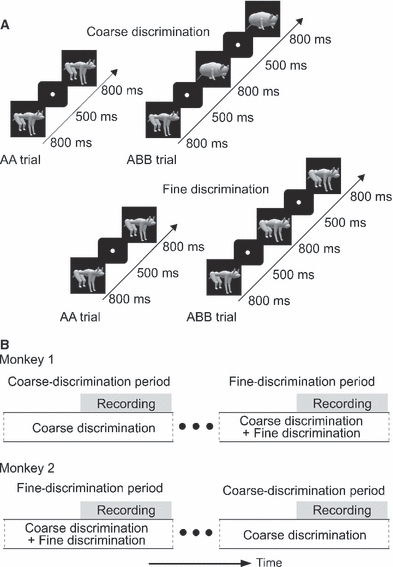
Behavioral task (A) and experimental procedure (B). (A) The task began with central fixation after which the first stimulus appeared for 800 ms. After a 500-ms delay, a second stimulus appeared. When the second stimulus was identical to the first stimulus (AA trial), the monkey had to release the lever. When the two stimuli were different, the monkey had to continue pressing the lever and wait for another 500 ms until a third stimulus appeared. The third stimulus was always identical to the second stimulus and thus the monkey had to release the lever (ABB trial). When the second stimulus was different from the first stimulus, it was selected from a group other than that of the first stimulus (coarse discrimination) or from the same group (fine discrimination). (B) In Monkey 1, the recording of neuronal activity was first conducted while the monkey was only experiencing the coarse discrimination (coarse-discrimination period) and then while the monkey was experiencing both the coarse and fine discrimination (fine-discrimination period). The order of recordings in the two conditions was reversed in Monkey 2. Note that, while the monkeys experienced both the fine and coarse discriminations in the fine-discrimination period, we consistently analyzed the responses recorded during the task of coarse discrimination for both groups of cells recorded in the coarse- and fine-discrimination periods.

The requirement of the task was to respond to a repetition of identical stimuli (Fig. 2A). The task started when the monkey pressed a lever. Following eye fixation on a fixation spot (0.5° in diameter) at the center of the screen for 900 ms, the first stimulus appeared for 800 ms. After a blank period of 500 ms only with the fixation spot, a second stimulus appeared for 800 ms. When the second stimulus was identical to the first stimulus, the monkey had to release the lever (‘AA trials’). Monkey 1 was allowed to respond immediately after the onset of the second stimulus, whereas Monkey 2 had to withhold the lever response until 500 ms after the onset of the second stimulus. The response had to be made within 1300 ms of the onset of the second stimulus in both monkeys. When the second stimulus was different from the first stimulus, the monkey had to keep pressing the lever until a third stimulus appeared after another blank period of 500 ms. The third stimulus was always identical to the second stimulus, and thus the subject always had to release the lever (‘ABB trials’). A drop of water was given after a correct response. The monkey could start the next trial at any time after an inter-trial interval of 1000 ms in Monkey 1 and 500 ms in Monkey 2. Eye fixation was required until the lever release. When the monkey made an incorrect response or broke the eye fixation, the trial was aborted, and a visual error signal (a green circle of 3.5° in diameter) was presented for 1000 ms, after which the inter-trial interval started. AA trials and ABB trials were intermixed in a quasi-random order. The first stimulus was quasi-randomly selected from the stimulus set. The second stimulus in ABB trials was also quasi-randomly selected from the stimulus set but following the rule described below.

In coarse-discrimination trials, the second stimulus was either a repetition of the first stimulus or selected from a group other than that of the first stimulus. Thus, the monkeys only needed to discriminate a repetition of an identical stimulus from a change across groups. In fine-discrimination trials, the second stimulus was either a repetition of the first stimulus or newly selected from the same group as the first stimulus. The monkeys had to discriminate a repetition of an identical stimulus from a subtle change between members within a group.

Both monkeys were first trained to conduct the task with images of natural objects, which were very different from one another. We then introduced the set of nine object stimuli and let the monkeys learn the coarse discrimination. Both monkeys reached > 85% correct responses within 2 months. Monkey 1 continued to conduct only the coarse discrimination, while we conducted the first series of neuronal activity recordings from the inferotemporal cortex (‘coarse-discrimination period’, Fig. 2B). After the first series of recordings were completed, Monkey 1 was trained for the fine discrimination. It took 4 months to reach > 75% correct responses. A second series of recordings from the inferotemporal cortex then started (Fig. 2B). Coarse-discrimination blocks as well as fine-discrimination blocks were provided to the monkey throughout the period of fine-discrimination training and the second series of recordings. We call this the ‘fine-discrimination period’ although we provided both fine- and coarse-discrimination blocks during this period. We recorded neuronal activities in both fine- and coarse-discrimination blocks, but we focus here on those obtained during the coarse-discrimination blocks. By doing so, the two sets of data obtained from the first and second series of recordings are only different from each other in the history of training, but not in the task content at the time of the recordings.

Monkey 2 moved to the training for the fine discrimination immediately after learning the coarse discrimination. It took 3 months for this monkey to reach > 70% correct responses on fine-discrimination trials. We then conducted the first series of recordings during the fine-discrimination period (Fig. 2B). The monkey experienced both coarse- and fine-discrimination blocks during this period, but we focus on neuronal data obtained during the coarse-discrimination blocks. After the first series of recordings were completed, we removed the fine-discrimination blocks and the monkey experienced only the coarse discrimination for 3 months. We then started the second series of recordings (Fig. 2B). We reversed the order of the fine- and coarse-discrimination periods from that of Monkey 1 so that the potential effects of time order or simple exposure to the stimulus images were opposite between the two monkeys. Monkey 1 was more exposed to the stimuli before the recordings in the fine-discrimination period than before the recordings in the coarse discrimination, whereas the opposite was the case in Monkey 2.

### Single-cell recordings

**Fig. 3:**
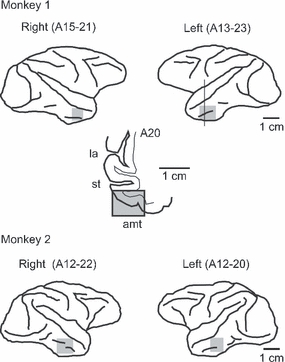
Recording positions. Activity of cells was recorded from the anterior part of the inferotemporal cortex, in the posterior/anterior range between 12 and 23 mm anterior to the ear bar position. The recordings were limited to the cortical extent on the ventrolateral surface of the brain, from the ventral lip of the superior temporal sulcus to the medial bank of the anterior middle temporal sulcus. The recording positions are shown by shading on the lateral views of the hemispheres. A ventrolateral part of the frontal section of the left hemisphere of Monkey 1 at anterior 20 is also shown in the middle. la, lateral fissure; st, superior temporal sulcus; amt, anterior middle temporal sulcus.

Extracellular recordings of action potentials of single neurons were made from the anterior part of the inferotemporal cortex, using the same experimental procedures as those described previously (Suzuki *et al.*, 2006). After taking magnetic resonance images of the monkey's brain, a head holder and recording chamber were implanted on the dorsal surface of the skull in an aseptic surgery under anesthesia with sodium pentobarbital (35 mg/kg followed by 10 mg/kg when necessary). Recordings were conducted with tungsten electrodes (FHC, Bowdoinham, ME, USA), which were guided by a guide tube and advanced by an Evarts-type manipulator (Narishige, Tokyo, Japan). The positions of recorded cells were determined with reference to the magnetic resonance images. Cells were recorded from the cortical extent on the ventrolateral surface of the brain, from the ventral lip of the superior temporal sulcus to the medial bank of the anterior middle temporal sulcus, in the posterior/anterior range between 12 and 23 mm anterior to the ear bar position (Fig. 3). Action potentials of single cells were isolated online using a template-matching method (Multi Spike Detector (MSD); Alpha Omega, Nazareth, Israel). If an isolated cell did not appear to be responding to any of the nine stimuli, we advanced the electrode to another cell. After all of the recordings were completed, the monkey was deeply anesthetized with a lethal intravenous dose of sodium pentobarbital (60–80 mg/kg) and perfused transcardially, the brain was removed, and brain slices were cut at 50 μm and stained for Nissl. We observed traces of the guide tubes and electrodes in the stained sections to confirm the estimated positions of the electrode tracks.

### Data analyses

We focused on neuronal responses to the first stimulus presentation in each trial to avoid the effects of preceding stimuli and of the monkey's decision. Only responses in correct trials were included. We analyzed only the cells for which any of the nine object stimuli was presented at least eight times as the first stimulus in the coarse-discrimination block.

**Fig. 4:**
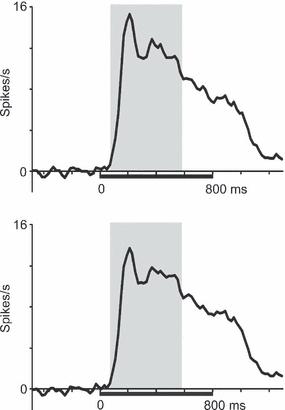
Time-course of averaged responses. First, responses to each stimulus were averaged across trials in individual cells. Then, the difference between the maximum and minimum responses among those to the nine stimuli was averaged over all the responsive cells that showed significant responses to at least one stimulus. The significance of responses was examined by comparing the discharges in the response window from 80 to 580 ms after the first stimulus onset (upper graph) or those in the window from 80 to 880 ms after the first stimulus onset (lower graph) with the discharges in the 500-ms window immediately before the first stimulus onset. The number of cells included in the averaging was 307 for the window from 80 to 580 ms (164 cells from Monkey 1 and 143 cells from Monkey 2), and 343 for the window from 80 to 880 ms (191 cells from Monkey 1 and 152 cells from Monkey 2). The shading indicates the window from 80 to 580 ms.

The significance of the response to each stimulus in each cell was examined by comparing the mean firing rate in the window from 80 to 580 ms after the onset of the first stimulus with the mean spontaneous firing rate averaged over the 500-ms period immediately before the first stimulus onset by paired *t*-test. The time window for responses was determined based on the response time of Monkey 1 and the time-course of grand average responses of responsive cells. The response time of correct bar release to the second stimulus presentation (in AA trials) in Monkey 1 was 468 ± 48 (mean ± SD) ms in the coarse-discrimination period, 525 ± 32 ms during the coarse-discrimination task and 522 ± 34 ms during the fine-discrimination task in the fine-discrimination period. This suggests that the first 500 ms was enough for the monkey to judge whether the second stimulus was identical to the first stimulus. The response time of Monkey 2 was longer (948 ± 48 ms in the coarse-discrimination period, 965 ± 66 ms during the coarse-discrimination task and 962 ± 62 ms during the fine-discrimination task in the fine-discrimination period), but this might be due to the constraint that we gave to this monkey (the release should be at least 500 ms after the stimulus onset). Also, the averaged responses (maximum–minimum) of responsive cells started to decline after around 580 ms (Fig. 4). Based on these observations, we decided to set the end of the analysis window at 580 ms after the stimulus onset. The beginning of the window was set at 80 ms because the averaged responses start to rise from the baseline at around 80 ms (Fig. 4). A cell was put into further analyses when its responses to at least one stimulus were significant [*P* < 0.05 after Bonferroni's correction for multiple comparison (*P* < 0.006)].

The magnitude of responses was represented by the mean firing rate in the window from 80 to 580 ms after the onset of the first stimulus. For the calculation of the half-width, kurtosis and ratio of the maximum among the responses to the apex stimuli to the overall maximum response (see below), we subtracted the mean spontaneous firing rate in the 500-ms period immediately before the first stimulus onset from the responses. For the other analyses (sparseness, tuning curve plot, consistency of response decrease direction), the original mean firing rate in the response window was used.

#### Sharpness of selectivity

**Fig. 5:**
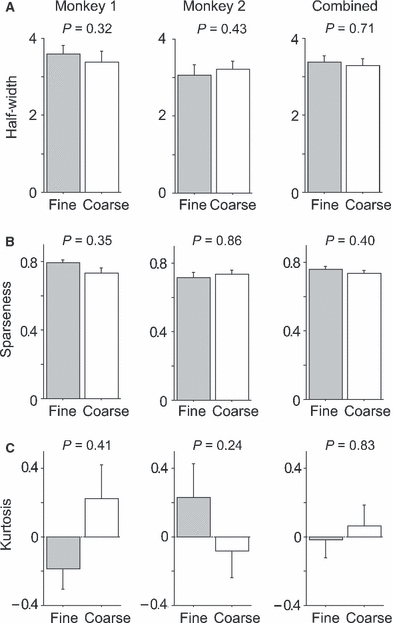
Comparison of half-width (A), sparseness (B) and kurtosis (C) representing selectivity sharpness between two groups of inferotemporal cells recorded in the fine- and coarse-discrimination periods. The error bars represent the SEM.

The sharpness of the stimulus selectivity of each cell was quantified by three measures (Fig. 5). The first measure was the number of stimuli that evoked a response of > 50% of the maximum response of the cell (half-width). It was determined to the first digit by interpolating responses just above and below 50% of the maximum response.

The second measure of selectivity sharpness was the sparseness defined by 
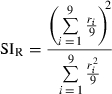
(1) where *r*_i_ represents the magnitude of the response to the *i*-th stimulus (Rolls & Tovee, 1995; Olshausen & Field, 2004). The value ranges from 1.0 to 0.11 in the case of nine stimuli, and smaller values indicate a greater sparseness or sharper selectivity.

The third measure was the kurtosis, which is popular in the sparse coding modeling literature (e.g. Olshausen & Field, 2004). It is defined by 
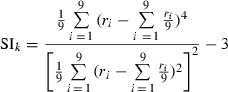
(2) where *r*_*i*_ represents the magnitude of response to the *i*-th stimulus. A larger value indicates a higher sparseness in this measure.

**Fig. 6:**
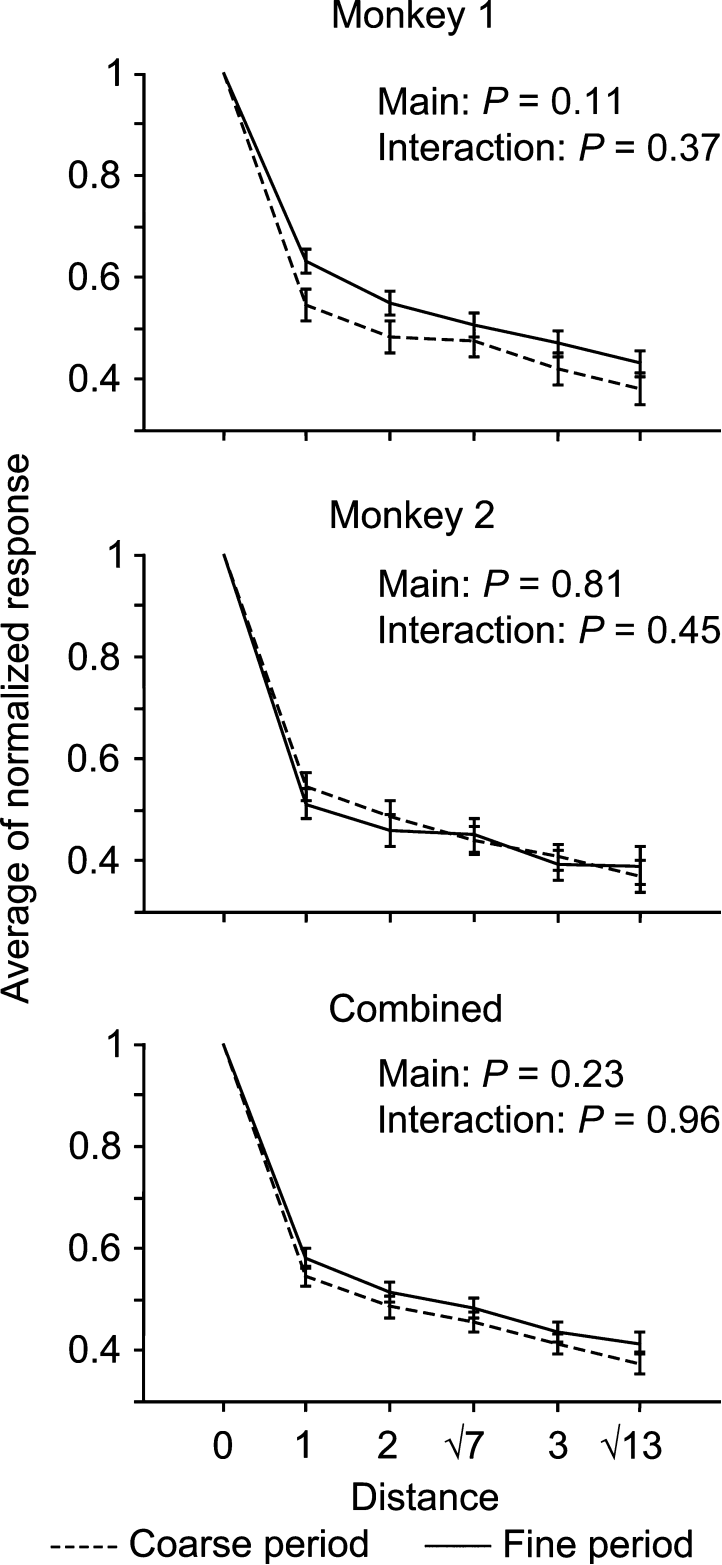
Comparison of tuning curves between two groups of inferotemporal cells recorded in the fine- and coarse-discrimination periods. Responses are plotted against distance of the stimulus from the position of the best stimulus in the parameter space. The responses were first normalized by the maximum response of each cell, and then averaged across cells at each distance. The distance between stimuli within each group was used as the unit of distance. The error bars represent the SEM.

The differences in the stimulus selectivity between the two periods were also examined by plotting responses against the distance of the stimulus from the best stimulus for each cell in the parametric space (tuning curve plot, Fig. 6). Because we roughly equalized the perceptual distances among the three apex objects, we assumed in this analysis that the three sides of the triangle had an identical length. There were two sets of distances, one for cells with the maximum response at an apex and the other for cells with the maximum response at a side position. We interpolated the points linearly to have values from all of the cells at all of these distances, except the largest distance for which responses were available only for cells with the maximum response at an apex. The responses of individual cells were normalized by the maximum response of the cell, and then averaged across cells at each distance. We applied a two-way repeated-measures anova (with period and distance as within-subject factors).

#### Onset latency of maximum responses in individual cells

The latency of response onset was determined for the maximum response of each cell. A peristimulus time histogram was constructed with 1-ms bins and smoothed with a Gaussian kernel (SD, 3 ms). The onset was defined by the first 1-ms bin of 20 consecutive bins that exceeded the mean of the spontaneous activity by more than 2.58 SD of the spontaneous activity. The mean and SD of the spontaneous activity were calculated for the 500-ms period immediately before the onset of the first stimulus, with the same Gaussian kernel for the SD.

#### Monotonicity of tuning

**Fig. 7:**
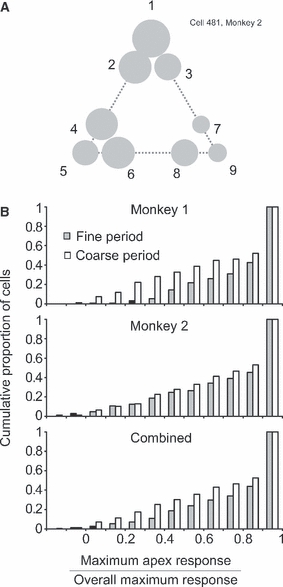
Monotonic tuning of responses along the axes in the parameter space. (A) Responses of an example inferotemporal cell recorded during the fine-discrimination period. The size of circles indicates the magnitude of the response evoked by the stimulus at the location. (B) Distribution of the ratio of the maximum among the responses to the three apex stimuli to the maximum response in each cell. The spontaneous activity of the cell was subtracted from both the numerator and denominator before calculating the ratio. The ordinate represents the accumulated number of cells converted to the proportion.

To compare the monotonicity of responses along axes in the parametric space between the fine- and coarse-discrimination periods, we first determined the maximum among the responses to the apex stimuli and divided it by the overall maximum response within each cell. This ratio would be 1 if the cell responded maximally to one of the apex stimuli. The distribution of the ratio across cells was compared between the two periods (Fig. 7).

**Fig. 8:**
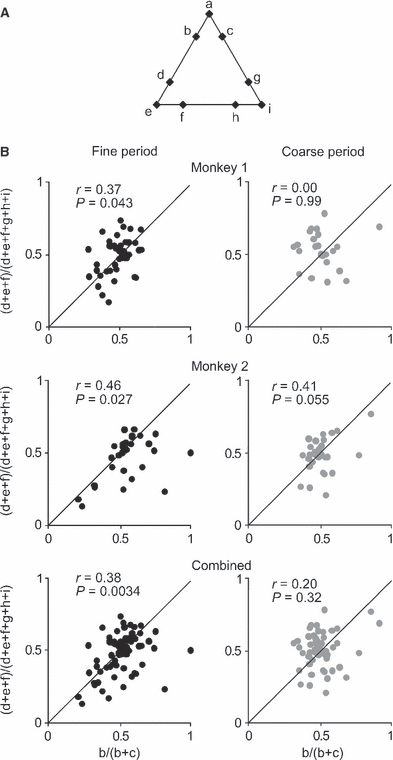
Consistency of differences in responses to stimuli along the two sides of the apex stimulus that evoked the maximum response. (A) The triangular configuration of the stimuli was rotated to place the best apex stimulus at the top (a). (B) The *x* value of a dot represents the magnitude of response to stimulus *b* divided by a sum of responses to stimuli *b* and *c*. The *y* value of a dot represents the averaged magnitude of responses to stimuli *d*, *e* and *f* divided by the averaged magnitude of responses to stimuli *d*, *e*, *f*, *g*, *h* and *i*. Only the 130 cells that maximally responded to one of the three apex stimuli are included in this analysis. The left graphs plot 74 cells recorded in the fine-discrimination period (44 in Monkey 1 and 30 in Monkey 2), and the right graphs plot 56 cells recorded in the coarse-discrimination period (24 in Monkey 1 and 32 in Monkey 2).

Secondly, for the cells that maximally responded to one of the three apex stimuli, we examined the consistency of selectivity between the two sides of the apex (Fig. 8). For simplicity of explanation, let us rotate the stimulus triangle to place the best stimulus at the top (a in Fig. 8A). The balance between the averaged responses to the left-bottom and right-bottom groups, (*r*_*d*_ + *r*_*e*_ + *r*_*f*_)/(*r*_*d*_ + *r*_*e*_ + *r*_*f*_ + *r*_*g*_ + *r*_*h*_ + *r*_*i*_), was plotted against the balance between the responses to the other two stimuli in the top group, *r*_*b*_/(*r*_*b*_ + *r*_*c*_). If the direction of the steepest response reduction is consistent in the parameter space, the two ratios should be positively correlated.

#### Reconstruction of stimulus arrangement from response patterns

**Fig. 9:**
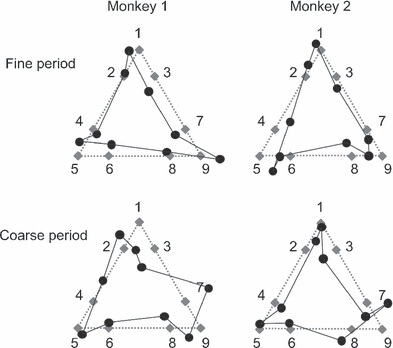
The configurations of the nine stimuli reconstructed from response patterns over the cell populations recorded during the fine-discrimination (top) and coarse-discrimination (bottom) period. The original dimension of the response space was the number of responsive cells recorded during the period, but it was reduced to two by principal component analysis.

Finally, we analyzed the representation of the stimuli by the combination of responses in the cell population (Fig. 9). We plotted the nine stimuli in the response space spanned by responses of all of the responsive neurons recorded during the period. Each dimension of the space represents responses evoked by the stimuli in one cell. The magnitudes of responses were normalized, in individual cells, by taking *z*-scores. More concretely, responses to each of the nine stimuli were first averaged across trials. The mean and SD were then calculated from the nine averaged responses. Finally, the difference of each averaged response from the mean was divided by the SD to obtain the normalized magnitude of response to each stimulus. The number of dimensions of the response space was reduced to two using principal component analysis.

## Results

### Behavioral data

The monkeys mastered the coarse discrimination within a relatively short period (2 months), whereas it took a longer time (4 months for Monkey 1 and 3 months for Monkey 2) to improve the performance in the fine discrimination. Even after the long learning period for fine discrimination, the monkeys made more errors in the fine discrimination than in the coarse discrimination (98 vs. 82% in Monkey 1 and 89 vs. 74% in Monkey 2). We estimated *d*′ for each pair of stimuli based on hits (keep holding in ABB trials) and false alarms (keep holding in AA trials) (MacMillan & Creelman, 1991). The data from coarse- and fine-discrimination blocks were combined for this purpose. The values of *d*′ were well correlated with the distances between the stimuli in the parameter space (correlation coefficients: 0.85 for Monkey 1 and 0.86 for Monkey 2). This high correlation indicates that the difficulty in discrimination quantitatively reflected the distances between stimuli in the parameter space even after the extensive training.

### Single-cell responses

We recorded the responses of 392 cells in the coarse-discrimination period (182 cells in Monkey 1 and 210 cells in Monkey 2) and 370 cells in the fine-discrimination period (233 cells in Monkey 1 and 137 cells in Monkey 2) (Fig. 2) from the anterior part of the inferotemporal cortex (Fig. 3). We analyzed their responses to the first stimulus presentation of each trial during the task. Among the 762 cells, a significant excitatory response (*P* < 0.05 by paired *t*-test after Bonferonni correction for multiple comparison) to at least one of the nine stimuli was seen in 157 cells recorded in the fine-discrimination period (93 cells in Monkey 1 and 64 cells in Monkey 2) and 150 cells recorded in the coarse-discrimination period (71 cells in Monkey 1 and 79 cells in Monkey 2). The results described below are based on these 157 and 150 cells. As described above, they are independent groups of cells recorded at different times.

Note that we consistently used responses recorded in blocks of trials in which the monkeys conducted the coarse discrimination. We included such blocks even in the fine-discrimination period. Therefore, responses recorded from the coarse- and fine-discrimination periods were taken in the same task but in different training contexts. This design enabled us to isolate the effects of learning context from other factors.

#### Sharpness of selectivity

We first compared the sharpness of stimulus selectivity between the two groups of inferotemporal cells recorded in the coarse- and fine-discrimination periods. We used half-width (the number of stimuli that evoked a response > 50% of the maximum response), sparseness and kurtosis to quantify the sharpness (see Materials and methods). None of them showed significant differences between the two groups of cells (half-width, *P*= 0.32 for Monkey 1, *P*= 0.43 for Monkey 2 and *P*= 0.71 for the combined data; sparseness, *P*= 0.35 for Monkey 1, *P*= 0.86 for Monkey 2 and *P*= 0.40 for the combined data; kurtosis, *P*= 0.41 for Monkey 1, *P*= 0.24 for Monkey 2 and *P*= 0.83 for the combined data by Mann–Whitney *U*-test, Fig. 5). The tuning curve plotted against the distance of the stimuli from the best stimulus in the parameter space also did not show significant differences between the two groups of cells (*P*= 0.11 for main effect of period and *P*= 0.37 for interaction for Monkey 1, *P*= 0.81 for main effect of period and *P*= 0.45 for interaction for Monkey 2, *P*= 0.23 for main effect of period and *P*= 0.96 for interaction for the combined data, Fig. 6). In summary, we did not find a tendency for the stimulus selectivity to be sharper in the period after long-term fine-discrimination training.

#### Response onset latency

We determined the latency of response onset for the maximum responses of individual cells and compared it between the two groups of cells. There were no significant differences between the cells recorded in the coarse- and fine-discrimination periods [138 ± 49 ms (mean ± SD) in the coarse-discrimination period vs. 136 ± 65 ms in the fine-discrimination period, *P*= 0.26 by Mann–Whitney test in Monkey 1; 141 ± 53 ms in the coarse-discrimination period vs. 125 ± 36 ms in the fine-discrimination period, *P*= 0.17 in Monkey 2; 140 ± 51 ms in the coarse-discrimination period vs. 132 ± 55 ms in the fine-discrimination period, *P*= 0.086 for the combined data].

#### Monotonicity of tuning

We then examined the monotonicity of tuning along axes of the parameter space. In the fine-discrimination period, there were more cells that responded maximally to one of the three apex stimuli than to the other stimuli. Figure 7A shows responses of one example cell recorded during the fine-discrimination period. The cell responded maximally to stimulus 1. Combining data from the two monkeys, the distribution of the ratio of the maximum among the responses to the apex stimuli to the overall maximum response in the fine-discrimination period was significantly more biased to larger values than the distribution in the coarse-discrimination period (*P*= 0.027 by Mann–Whitney *U*-test, Fig. 7B). When we separated the data from the two monkeys, there were differences of consistent direction in both monkeys, although the difference reached significance only in Monkey 1 (*P*= 0.026 in Monkey 1 and *P*= 0.41 in Monkey 2).

To further examine the monotonicity of tuning with reference to the parameter space, we examined the consistency of the response difference between the two sides of the apex that evoked the maximum response. The cell exemplified in Fig. 7A showed larger responses to stimuli positioned in the counter-clockwise direction from stimulus 1 (stimuli 2, 4, 5 and 6) than to the stimuli on the other side (stimuli 3, 7, 9 and 8). For the cells that responded maximally to one of the apex stimuli, the ratio between the averaged responses to the two other groups of stimuli and that of the best stimulus was plotted against the ratio between responses to the other stimuli that belonged to the same group as the best stimulus (Fig. 8). If the direction of the steepest response reduction is consistent in the parameter space, the ratio should be similar between the two comparisons. There was a significant positive correlation for the cells recorded in the fine-discrimination period (*P*= 0.0034), whereas there was no significant correlation for the cells recorded in the coarse-discrimination period (*P*= 0.32). The same was true in either monkey when the data obtained from each monkey were analyzed separately (*P*= 0.043 and *P*= 0.999 in the fine- and coarse-discrimination periods, respectively for Monkey 1; *P*= 0.027 and *P*= 0.055 in the fine- and coarse-discrimination periods, respectively for Monkey 2). Even when the other responsive cells, which maximally responded to one of the side stimuli, were included in the plot by placing the best stimulus at b or c in Fig. 8A, similar results were obtained (*P*= 0.011 in the fine-discrimination period and *P*= 0.23 in the coarse-discrimination period for the combined data, not shown). These results show that the responses of inferotemporal cells were more monotonically tuned with reference to the parameter space during the fine-discrimination period than during the coarse-discrimination period.

### Reconstruction of shape arrangement in response patterns

To examine how a combination of responses in a cell population faithfully represents the arrangement of stimuli in the parameter space, we plotted the nine stimuli in the response space spanned by the responses of all of the responsive cells recorded in the period. The number of dimensions of the space, which was originally the number of responsive cells recorded in the period, was reduced to two by principal component analysis. The arrangement of the nine stimuli reconstructed from the responses recorded during the fine-discrimination period appeared more similar to the original triangular shape than that reconstructed from the responses recorded during the coarse-discrimination period (Fig. 9). Quantitatively, the angles at the stimuli along the sides of the triangle were closer to 180° in the stimulus arrangement reconstructed from the fine-discrimination period [161 ± 19° (mean ± SD) for the 12 positions in two monkeys] than in the stimulus arrangement reconstructed from the coarse-discrimination period (134 ± 36°) (*P*= 0.037, paired *t-*test).

To find the relation between the monotonic tuning of individual cells and the faithful reconstruction of the stimulus arrangement in the response space of the cell population, we examined the degradation that occurred after removing 20 monotonic cells (those with a ratio of the maximum among the responses to the apex stimuli to the overall maximum response > 0.9) and compared it with the degradation that occurred by removing 20 non-monotonic cells (those with smaller ratios). We focused on the data obtained in the fine-discrimination period, and then the number of the responsive cells used for the original reconstruction was 93 in Monkey 1 and 64 in Monkey 2. The 20 cells to remove were randomly selected from each cell group and the random selection was repeated 100 times for each case. The removal of 20 monotonic cells degraded the arrangement more than the removal of 20 non-monotonic cells; the removal of 20 monotonic cells decreased the angles at the stimulus positions on the sides of the triangle more [from 164 ± 16° (mean ± SD) to 144 ± 11° in Monkey 1 and from 158 ± 22 to 107 ± 9° in Monkey 2] than the removal of 20 non-monotonic cells (from 164 ± 16 to 159 ± 2° in Monkey 1 and from 158 ± 22 to 149 ± 5° in Monkey 2) (*P*< 0.0001 in each monkey, *t*-test).

We also plotted the nine stimuli only based on the responses of 20 cells randomly selected from the monotonic cells recorded in the fine-discrimination period, reduced the space dimension to two, and compared the arrangement of the stimuli with that reconstructed only based on the responses of 20 cells randomly selected from the non-monotonic cells recorded in the same period. The random selection of 20 cells was repeated 100 times for each cell group. The arrangement of the nine stimuli reconstructed from the responses of monotonic cells was more similar to the original triangular shape than that reconstructed from the responses of non-monotonic cells; the angles at the stimuli along the sides of the triangle were closer to 180° in the stimulus arrangement reconstructed based on 20 monotonic cells [145 ± 11° (mean ± SD) in Monkey 1 and 133 ± 14° in Monkey 2] than in the stimulus arrangement reconstructed based on 20 non-monotonic cells (74 ± 14° in Monkey 1 and 70 ± 11° in Monkey 2) (*P*< 0.0001 in each monkey, *t*-test). These two sets of results suggest that the monotonic cells contributed more than the non-monotonic cells to the faithful reconstruction of the stimulus arrangement in the response space of the cell population.

## Discussion

To examine whether there are any neural correlates in the inferotemporal cortex for visual expertise of fine discrimination among similar objects, we recorded the responses of inferotemporal cells in two different training histories of monkeys. One group of recordings was conducted after the monkeys had been extensively trained for fine discrimination (fine-discrimination period), and the other group of recordings was conducted after the monkeys had been exposed only for coarse discrimination for several months (coarse-discrimination period). The training condition continued throughout the recording period. We did not find any evidence of sharper tuning in the fine-discrimination period, whereas we found that the tuning of responses was more monotonic in the fine-discrimination period. In the fine-discrimination period, stimuli located at the apexes of the triangle in the stimulus parameter space evoked the maximum or close-to-maximum responses in a larger proportion of cells, and the direction of response decrease in the parameter space was more consistent than in the coarse-discrimination period. Possibly due to this larger monotonicity in individual cells, the response patterns of cell populations recorded in the fine-discrimination period more faithfully represented the configuration of the shape stimuli in the parameter space.

These differences could not be caused by differences in the behavioral set of the monkeys, such as attention and motivation, because the data used in this study were recorded in the blocks composed only of coarse-discrimination trials. The coarse-discrimination blocks were alternated with the fine-discrimination blocks in the fine-discrimination period. In our previous study (Suzuki *et al.*, 2006), in which the same stimuli and tasks as those in the present study were used, we found that a switching between coarse- and fine-discrimination tasks within a daily session did not significantly change the tunings of inferotemporal cells. Thus, taken together, it is suggested that the response selectivities of inferotemporal cells change along long-term learning, but remain stable across changes in behavioral context within a day.

Monkey 1 had never experienced the fine discrimination before the coarse-discrimination period. Monkey 2 had once learned the fine discrimination, but had not experienced it for 3 months when the second group of recordings started. We let Monkey 2 go through the course opposite to that of Monkey 1 so as to discriminate potential effects of longer exposure to the stimuli from effects of fine-discrimination training. If the recordings during the fine-discrimination period had followed those in the coarse-discrimination period in both monkeys, we could not have discriminated the two effects from each other. As the data obtained from the two monkeys consistently showed larger monotonicity in the fine-discrimination period, which occurred after the coarse-discrimination period in Monkey 1 and before the coarse-discrimination period in Monkey 2, the larger monotonicity could not have been caused by the longer exposure to the stimuli.

In Monkey 2, the effects of the initial fine-discrimination training might persist into the coarse-discrimination period. It has been shown that the effects of perceptual learning remain for more than several months (Fiorentini & Berardi, 1981; Ball *et al.*, 1988; Karni & Sagi, 1993; Beard *et al.*, 1995; Sommerhalder *et al.*, 2003; Chung *et al.*, 2004). However, although a part of the effects remained, the magnitude of the effects more or less decayed in many of these previous studies (Fiorentini & Berardi, 1981; Karni & Sagi, 1993; Chung *et al.*, 2004). In Monkey 2, the effects of the initial fine-discrimination training should have been smaller, due to decay in the coarse-discrimination period, than those seen in the fine-discrimination period. Therefore, the differences in Monkey 2 should have been in the same direction as those seen in Monkey 1, although the magnitude of differences might be smaller in Monkey 2. In fact, this was the case in the present data with regard to both the magnitude of the maximum response to apex stimuli and the consistency of response decrease direction in the parameter space. Therefore, the present results and conclusions are consistent with the possibility of persistence in the effects of fine-discrimination training. The larger monotonicity of individual inferotemporal cells and the more faithful reconstruction of the configuration of stimuli were probably due to the fine-discrimination training immediately before and during the recordings.

Several recent studies, using a set of shape stimuli distributed in a limited range of parameter space, have shown that inferotemporal cells tend to show monotonic tuning of responses along axes in the parameter space (Kayaert *et al.*, 2005; Leopold *et al.*, 2006; De Baene *et al.*, 2007; Freiwald *et al.*, 2009). Although the types of stimuli varied from simple geometric shapes (Kayaert *et al.*, 2005) to intermediately complex geometric shapes (De Baene *et al.*, 2007) to realistic and caricature human face models (Leopold *et al.*, 2006; Freiwald *et al.*, 2009), the stimuli within the set were similar to one another. Unique to the present study, we found that the monotonic tuning developed with fine-discrimination training.

Two mechanisms have been proposed for perceptual learning. One is changes in neuronal selectivity in the sensory cortical areas where the relevant features of stimuli are represented (Gilbert *et al.*, 2001; Schoups *et al.*, 2001; Ghose *et al.*, 2002; Yang & Maunsell, 2004; Raiguel *et al.*, 2006). The other is changes in the connectivity between the sensory areas and decision sites, or the way by which the decision site decodes signals from the sensory areas (Chowdhury & DeAngelis, 2008; Law & Gold, 2008). For example, the decision site may learn to focus on outputs from the cells in sensory areas that are most useful for the task. The present results show that the monkeys’ visual expertise for the discrimination of similar complex shapes may be at least partly based on changes in the selectivity of cells in the sensory side, in our case the inferotemporal cortex.

There are at least two advantages for the monotonic tuning of responses. First, the cells with monotonic tuning can contribute to discrimination between all pairs of stimuli except those aligned along the axis orthogonal to the main axis of the response change. A cell that has a response peak at a middle position in the parameter space provides confusing signals for the discrimination between two stimuli located on opposite sides of the peak. Although they are far from each other in the parameter space, the magnitudes of responses to the two stimuli are close to each other. Outputs from this cell have to be neglected in the discrimination of the two stimuli. Different sets of cells will have to be selected for the discrimination of different stimulus pairs. The representation in a cell population only composed of cells with monotonic tuning does not involve this complication. As the magnitude of response of a cell changes monotonically along an axis in the parameter axis, the magnitude of response to a given stimulus indicates the position of the stimulus along the axis. By reading the magnitudes of responses in multiple cells that are tuned for different axes in the parameter space, the position of the given stimulus can be uniquely determined. The minimum number of cells required for the representation is the same as the number of dimensions of the parameter space over which stimuli were distributed (two in the present experiment). By having more cells, the decoding will be more accurate, or more robust to noises.

The second advantage is a greater generalization of expertise to new examples (Guigon, 2003; Tanaka *et al.*, 2005). The capability to deal with new examples is a part of the visual expertise. In a system only composed of cells with monotonic tuning, the generalization to new stimuli is straightforward. Once the monotonic tuning of responses is established over the experienced stimuli, responses to new stimuli located between the experienced stimuli in the parameter space will also reflect the distances between the stimuli in the parameter space. The cells with peaks at middle positions may provide disturbance. With such cells included, new combinations of cells have to be newly learned for new stimulus pairs to obtain the best performance.

Owing to these advantages, the increase of inferotemporal cells with monotonic tunings may support the development of visual expertise. The first advantage of monotonic tuning partly demonstrates it in the faithful reconstruction of the triangular arrangement of stimuli. The neuronal responses recorded in the fine-discrimination period more faithfully reconstructed the original arrangement of the stimuli in the parameter space than the neuronal responses during the coarse-discrimination period. Because the arrangement of the nine stimuli reconstructed based on the responses of 20 randomly selected monotonic cells was more similar to the original arrangement than that reconstructed based on the responses of 20 randomly selected non-monotonic cells, the more faithful reconstruction was probably due to the larger percentage of monotonic cells in the fine-discrimination period. This notion was also supported by the finding that a removal of 20 randomly selected monotonic cells degraded the stimulus arrangement reconstructed based on responses of the whole responsive cells recorded in the fine-discrimination period more than a removal of 20 randomly selected non-monotonic cells. Previous studies have shown similar faithful reconstruction of the arrangement of complex shape stimuli from discrimination performance of monkeys or from responses of cells in the inferotemporal cortex (Sugihara *et al.*, 1998; Op de Beeck *et al.*, 2001; Sigala *et al.*, 2002). The present study has added the fact that monotonic responses contributed more to the faithful reconstruction than other more complicated responses. It also suggests that the faithful reconstruction developed through the experience of fine discrimination.

In conclusion, by using a set of parametrically designed object stimuli, we found that the responses of cells in the monkey inferotemporal cortex were more monotonically tuned in the stimulus parameter space after extensive training of the monkeys for fine discrimination among the stimuli. The proportion of such cells might have increased or their tuning might become more monotonic through the fine-discrimination training. It is suggested that the visual expertise in discriminating a particular group of objects is based on the development of inferotemporal cells with monotonic response tuning over the discriminated objects.
